# Molecular characterization of enterotoxin genes in methicillin-resistant *S. aureus* isolated from food poisoning outbreaks in Egypt

**DOI:** 10.1186/s41043-023-00416-z

**Published:** 2023-08-28

**Authors:** Heba A. Ramadan, Ahmed M. El-Baz, Reham M. Goda, Mohamed M. A. El-Sokkary, Rasha M. El-Morsi

**Affiliations:** 1 Department of Microbiology and Immunology, Faculty of Pharmacy, Delta University for Science and Technology, Gamasa, 11152 Egypt; 2 Microbiology and Immunology Department, Faculty of Pharmacy, Mansoura University, Mansoura, 35516 Egypt

**Keywords:** *Staphylococcus aureus*, Food poisoning, MRSA, Enterotoxin genes, Microarrays

## Abstract

**Background:**

*Staphylococcus aureus* (*S. aureus*), especially methicillin-resistant *S. aureus* (MRSA), is a known disease-causing bacteria with many associated health hazards. Staphylococcal food poisoning can result from staphylococcal enterotoxins (SEs).

**Methods:**

In this study, 50 *S. aureus* isolates were isolated from the gastrointestinal tract (GIT) clinical samples of patients with food poisoning in clinical laboratories at Mansoura University Hospital, Egypt. For determination their antibiogram, these isolates were tested for antimicrobial sensitivity against 12 antimicrobial agents using the agar disk diffusion test. After DNA extraction from the isolates, conventional polymerase chain reaction (PCR) was used to detect *mecA* and SEs genes.

**Results:**

As a result, all isolates were ampicillin and cefoxitin-resistant, while 86% (43 of 50) of the tested isolates exhibited multidrug resistance (MDR). In contrast, the highest sensitivity was confirmed against vancomycin, linezolid and quinolones, namely ciprofloxacin and norfloxacin. Although 100% of the isolates were *mecA* positive, staphylococcal enterotoxin genes *set-A*, *set-B, set-C, set-G, set-M,* and *set-O* genes were detected in 56%, 20%, 8%, 32%, 16%, and 24%, of the tested isolates, respectively. Finally, isolates encompassing SEs genes were used to validate a microarray chip, indicating its potential for a better methodological approach for detecting and identifying SEs in human samples.

**Conclusion:**

The genotypic findings of this study may help explain the enterotoxigenic patterns in *S. aureus* among Egyptian patients with food poisoning.

## Introduction

*S. aureus*, as a member of Gram-positive bacteria, could also be associated with a large-scale food poisoning outbreak, where staphylococcal enterotoxins (SEs) producing *S. aureus* were identified as the causative pathogen [1]. *S. aureus* produces many virulence factors, including proteases, enterotoxins, hemolysins, leukocidins, exfoliative toxins, and immune-modulatory factors [2]. The term ‘superantigens’ commonly refers to toxic-shock syndrome toxin-1 (TSST-1) and staphylococcal enterotoxins (SEs), produced by *S.aureus* activating a large population of specific T-cells at pictogram levels [3]. A large family of structurally related toxins, e.g., staphylococcal enterotoxins (SEs), are considered as one the most potent virulence factors, with the ability to stimulate T lymphocyte proliferation and induce the release of cytokines, and ultimately cause cell death [3]. Interestingly, plasmids, bacteriophages, and pathogenicity islets are capable of transporting genes encoding such toxins [4]. In staphylococcal food poisoning, a toxigenic process is triggered by a class of toxins known as SEs, primarily described in previous studies [5]. These toxins are associated with a form of gastroenteritis, characterized by vomiting and diarrhea [6]. Moreover, SEs play a role in rheumatoid arthritis [7], atopic eczema [8], and others [9]. SEs, as members of the superantigen protein family, can elicit a polyclonal immune response [10]. These toxins may also influence the host's immune response and contribute to bacterial persistence and defense [11]. *S. aureus* expression of particular enterotoxin (s*et*) genes depends on the source of the host tissue and may play a role in the adaptation of *S. aureus* to the host environment [12]. Most SEs are heat-stable and are not affected by digestive enzymes, with the ability to induce various symptoms, such as nausea, vomiting, malaise, abdominal cramps, and diarrhea [6]. Interestingly, about 95% of “staphylococcal” food poisoning outbreaks are caused by classical types of SEs, namely A, B, C, D, and E [13]. In addition, the consumption of food contaminated with enterotoxins produced by methicillin-resistant *S. aureus* (MRSA) could be associated with serious complications [14, 15] such as infection of heart valves (endocarditis), gangrene or death of the soft tissues (necrotizing fasciitis), and bone or joint infections (osteomyelitis or septic arthritis) [16–18]. Moreover, MRSA has wider economic implications that encompass indirect expenses to the patient and society, and also hospitalization costs [19]. For instance, compared to MSSA, MRSA results in an approximately threefold increase in expenses for primary nosocomial bloodstream infections [20].

Nearly twenty-seven types of SEs have been detected and identified as single-chain proteins, with a molecular weight ranging from 19 to 29 kD. Two major toxic activities that contribute to the effects of SEs have been identified: A neurotoxic activity, which stimulates vomiting reflexes by stimulating the brain's emetic center and vagus nerve, and a superantigenic activity, which produces strong T lymphocyte activation and severe fever [21].

Various techniques have been used to identify SEs. One of the traditional methods is serological typing, which is based on antigenic detection; however, it is only semi-quantitative and lacks specificity and sensitivity [22]. Other techniques, such as PCR and DNA-DNA hybridization, have been introduced recently. Nevertheless, these techniques are limited and can only detect one or a few toxins in one experiment [22, 23]. Moreover, multiplex PCR was used to detect several genes simultaneously. To achieve unambiguous identification of *set*-specific amplicons, additional restriction endonuclease tests or other procedures are required [24].

Consequently, a rapid and specific method for the simultaneous detection and identification of SEs for diagnostic and epidemiological purposes are still required. The molecular basis of relationships can be studied using microarrays on a scale that is not conceivable with traditional research. [25]. The primary objective of this study was to determine the relationship between the resistance of MRSA, isolated from Egyptian patients with food poisoning, and the presence of various types of enterotoxins. By validating an enterotoxin-specific microarray, a better methodological approach for identifying several enterotoxins in one experiment could be provided.

## Materials and methods

### Bacterial isolation and identification

In total, 50 *S. aureus* isolates were collected from 157 gastrointestinal tract (GIT) clinical samples (stool samples) of patients with food poisoning in the clinical laboratories of Mansoura University Hospital, Egypt, from June to September 2021. In the next step, each stool sample needed to be put into clean, dry plastic jars with screw-top lids and had to be brought to the lab right away. All isolates were identified using standard microbiological tests [26]. Swabs were inoculated onto nutrient agar plates and incubated aerobically at 37 °C for 24–48 h. The obtained colonies were identified as *S. aureus* by conventional laboratory identification methods, including colonial morphology, Gram stain, catalase test, coagulase test, and growth on mannitol salt agar (MSA) [26].

The collected isolates, 50 (31.8%) isolated *S. aureus* from 157 stool samples included 62% from males, while 34% of the total number of cases were < 19 years old, 40% of the cases were between the ages of 19 and 40 years, 12% of cases were between the ages of 41 and 60 years, and 14% of cases were > 60 years old.

#### Coagulase test

Using the coagulase tube approach, 500 µl of each isolate's overnight broth culture was combined with an equal amount of human plasma in sterile glass test tubes. The tubes were then incubated at 37 °C in an incubator or water bath and observed every 30 min. The tube is retained at room temperature for overnight incubation if the test is negative after four hours at 37 °C [26].

#### Catalase test

The test was carried out by adding a fresh pure colony from the overnight culture with a sterile tip and 1–2 drops of 3% hydrogen peroxide (H_2_O_2_) on a dry, clean slide [26].

### Antimicrobial susceptibility

Antimicrobial susceptibility was determined using the disc diffusion method, in accordance with the Clinical and Laboratory Standard Institute (CLSI) [27] for the following antibiotics: Ampicillin (AMP, 10 µg), cefoxitin (FOX, 30 µg), cefotaxime (CTX, 30 µg), ciprofloxacin (CIP, 5 µg), norfloxacin (NOR, 10 µg), tobramycin (TOB, 10 µg), gentamicin (GEN, 10 µg), doxycycline (DOX, 5 µg), amikacin (AMK, 30 µg), azithromycin (AZM, 15 µg), nitrofurantoin (NIT, 300 µg), amikacin, vancomycin (VA, 30 µg), linezolid (LZD, 30 µg) and sulfamethoxazole/trimethoprim (SXT, 1.25/23.75 µg) (Oxoid, Hampshire, England). The results were interpreted using the criteria outlined in CLSI guidelines based on the inhibition zone produced, which correlate with susceptibility levels [27]. The obtained results were used to identify the percentage of MDR among the tested isolates. As previously documented, multidrug resistance (MDR) is defined as resistance to three or more antimicrobial classes [28]. The phenotypic identification of the isolates as MRSA was performed against cefoxitin through the disk diffusion method, while the standard strain of *S. aureus* (ATCC 29312) was included as a control isolate.

### Total DNA extraction

Pure colonies of freshly grown cells were incubated at 37 °C in Mueller Hinton Broth (MHB) (Oxoid, Hampshire, England) for 24 h. Pellets were collected after centrifugation from a 2 mL culture of each isolate. In the next step, each resulting pellet was suspended in 180 µL lysozyme (10 mg/mL), as recommended for Gram-positive bacteria (Sigma-Aldrich Co., UK), in a lysate buffer. Chromosomal DNA was extracted using the Gene JET Genomic DNA purification Kit (Thermo Scientific, Vilnius, Lithuania), in accordance with the manufacturer's guidelines.

Genomic DNA concentration and purity were determined in the prepared samples by measuring absorbance at 260 and 280 nm using nanodrop instrument (OptizenNanoQ, Daejeon, Korea).

### Conventional PCR amplification for the detection of different staphylococcal enterotoxin and mecA genes

Various *set* and *mecA* genes were screened using the primers listed in Table 1. Each PCR reaction contained 12.5 µL of DreamTaq Green PCR Master Mix (Thermo Scientific, Vilnius, Lithuania), 1µL (10 µM) of each primer, 2 µL of template DNA, and up to 25 µL of nuclease-free water. Each PCR reaction started with an initial denaturation at 94 °C for 5 min, followed by 35 cycles of three-step denaturation at 94 °C for 30 s, annealing temperature (Table 1) for 30 s, and extension at 72 °C for 45 s. Finally, each PCR reaction was terminated with a final extension at 72 °C for 5 min. The resulting PCR products and the GeneRuler 100 bp plus DNA ladder (Thermo Fischer Scientific, USA) were separated on a 1.5% agarose gel and then stained with ethidium bromide (Merck, Hohenbrunn, Germany). The produced amplification bands were photographed using a UV transilluminatorUV-TM-25–230 v (Hoefer Inc.). Cluster analysis for *set* genes carrying isolates was performed using the un-weighted pair-group method with average linkages (UPGMA).

**Table 1:** Primers used in this work to detect *mecA* and Staphylococcal enterotoxin genes

Primer name	Primer sequence	Annealing temp. (°C)	Size (bp)	Citations
*Mec-A*				
F	GTAGAAATGACTGAACGTCCGATAA	52	310	[29]
R	CCAATTCCACATTGTTCGGTCTAA			
*Set-A*				
F	GGATATTGTTGATAAATATAAAGGGAAAAAAG	53	439	[30]
R	GTTAATCGTTTTATTATCTCTATATATTCTTAATAGT			
Oligo	GTTTCATACTTCTACAGAACCTTC			
*Set-B*				
F	AGATTTAGCTGATAAATACAAAGATAAATACG	54	494	[30]
R	TCGTAAGATAAACTTCAATCTTCACATCT			
Oligo	AAACTCTATGAATTTAACAACTCG			
*Set-C*				
F	AGATTTAGCAAAGAAGTACAAAGATG	53	490	[30]
R	AAGGTGGACTTCTATCTTCACACTT			
Oligo	AACCACTTTGATAATGGGAACTTA			
*Set-D*				
F	AGATTTAGCAAAGAAGTACAAAGATG	53	481	[30]
R	CTACTTTTCATATAAATAGATGTCAATATG			
Oligo	TCAATTTGTGGATAAATGGTGTAC			
*Set-E*				
F	AGATTTAGCAAAGAAGTACAAAGATG	54	473	[30]
R	TGTATAAATACAAATCAATATGGAGGTTCTCT			
Oligo	TGGTTTATATAACTCAGACAGCTTT			
*Set-G*				
F	AGAATTAGCTAACAATTATAAAGATAAAAAAG	52	496	[30]
R	TCAGTGAGTATTAAGAAATACTTCCAT			
Oligo	AACAATCGACAATAGACAATCACT			
*Set-I*				
F	TGATTTAGCTCAGAAGTTTAAAAATAAAAATG	52	505	[30]
R	TTAGTTACTATCTACATATGATATTTCGA			
Oligo	AGGCAAAGAATATGGATATAAATCT			
*Set-M*				
F	ATGAAAAGAATACTTATCATTGTTGTTTTATTG	53	720	[30]
R	CTTCAACTTTCGTCCTTATAAGATATTTC			
Oligo	AACAGGACAAGCTGAAAGTTTC			
*Set-N*				
F	ATAAAAAATATTAAAAAGCTTATGAGATTGTTC	51	777	[30]
R	ACTTAATCTTTATATAAAAATACATCAATATG			
Oligo	ATACAATAAAGATACCGGTAACAT			
*Set-O*				
F	TATGTAGTGTAAACAATGCATATGCA	53	685	[30]
R	TCTATTGTTTTATTATCATTATAAATTTGCAAAT			
Oligo	ATGACAGAATGACTAGTGATGTA			

### Oligo array printing

**Fig. 1:**
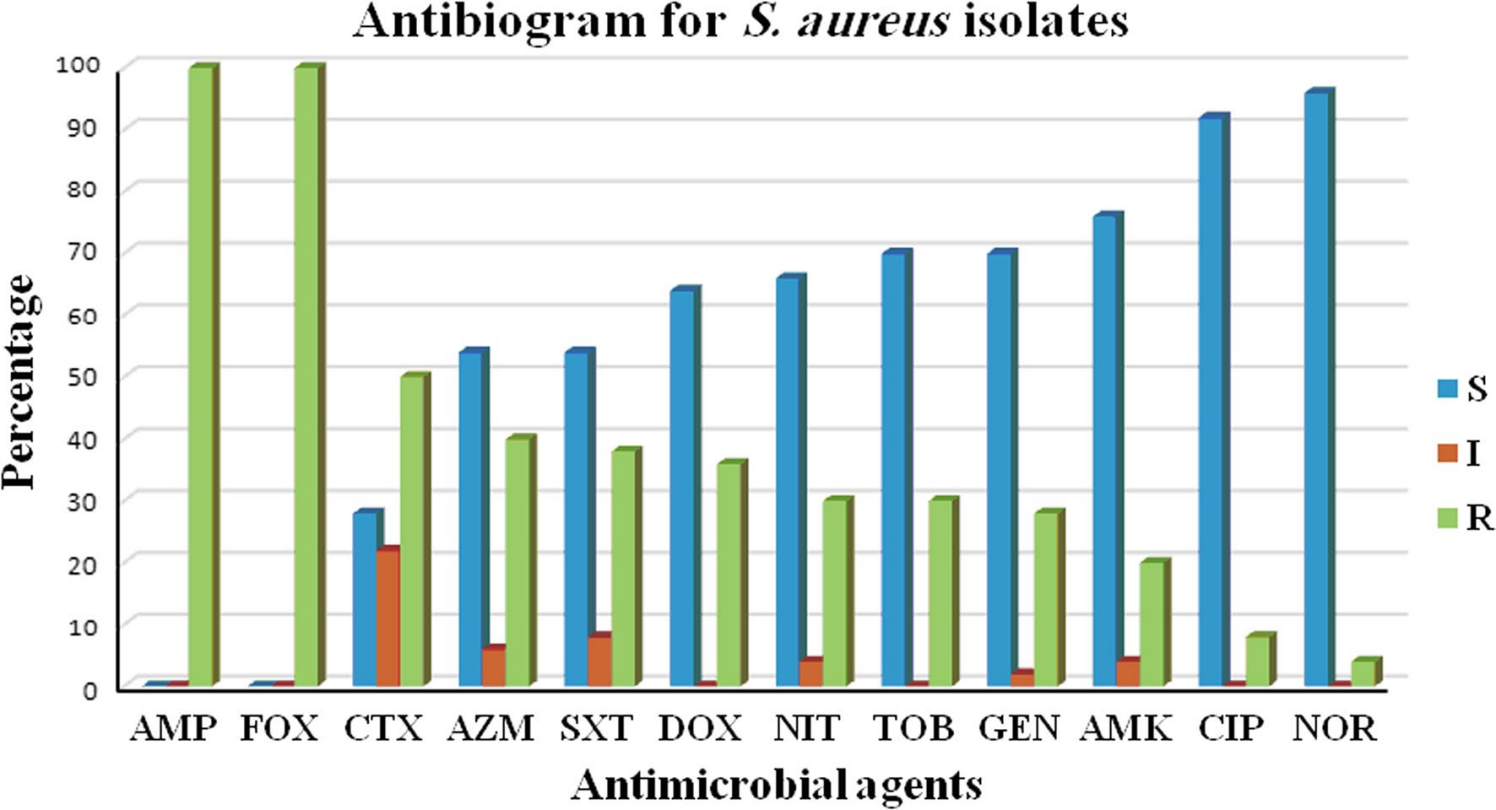
Antibiogram for *S. aureus* isolates against 12 antimicrobial agents, S: sensitive, I: intermediate resistant, R: resistant

Microarray slides (Scienion, Berlin, Germany) were spotted in triplicate for each probe using The SpotBot microarrayer (ArrayIt, Sunnyvale, California, USA) spotting machine (Fig. 1). Each oligonucleotide solution was prepared using ArrayIt spotting buffer (ArrayIt, Sunnyvale, California, USA) in a 384-well printing plate.

### Microarray testing

Genomic DNA samples were labeled (Thermo Fisher Scientific, Waltham, Massachusetts, USA) and then resuspended in the hybridization buffer (Scienion, Berlin, Germany). Hybridizations were carried out in a hybridization station (ArrayIt, Sunnyvale, California, USA). After hybridization step, microarray slides were then washed using buffers I, II, and III (Scienion, Berlin, Germany). Colorimetric observation was performed after staining using streptavidin–biotin color development system (ThermoFisher Scientific, Waltham, Massachusetts, USA), following the manufacturer’s instructions. Finally, images were acquired using the ArrayIt Microarray Scanner (ArrayIt, Sunnyvale, California, USA). Signal intensities were recorded using the Spotware software (ArrayIt, Sunnyvale, California, USA). Final intensities were calculated after subtracting the local background values from the per-sample median values.

## Results

### Identification of S. aureus isolates

A total of 50 (31.8%) unique *S. aureus* clinical isolates were separated from 157 stool samples, and identified in the current study. Most cases (62%) were males, while 34% of the total number of cases were < 19 years old, 40% of the cases were between the ages of 19 and 40 years, 12% of cases were between the ages of 41 and 60 years, and 14% of cases were > 60 years old. After growth on nutrient agar, *S. aureus* isolates appeared as colonies of a golden yellow hue. Gram staining indicated positive type for all isolated bacteria exhibiting a grape-like arrangement under the microscope. All the tested isolates were given immediate effervescent with 3% hydrogen peroxide. In addition, they were coagulase positive and induced gelling of the plasma. The tested isolates had the ability to ferment mannitol biochemically with yellow color produced on mannitol salt agar medium (MSA).

### Antimicrobial susceptibility testing

Antimicrobial susceptibility testing revealed that all isolates were ampicillin- and cefoxitin-resistant (100%), whereas 72% were cefotaxime-resistant. In contrast, the highest sensitivity was confirmed against vancomycin, linezolid, where all isolates did not exhibit any resistance to the tested antibiotics. Similarly, there was seldom any observed resistance against ciprofloxacin (8%) and norfloxacin (4%). Other antimicrobials such as azithromycin (46%), sulfamethoxazole/trimethoprim (46%), doxycycline (36%), nitrofurantoin (34%), tobramycin (30%), gentamicin (30%), and amikacin (24%) exhibited intermediate resistance (Table 2 and Fig. 1). In this study, 86% (43 of 50) of all isolates exhibited MDR, with resistance to more than 2 classes of antimicrobial agents. Patterns P1 (referred to as bacterial isolates resistant to 2 antimicrobial agents; FOX and AM) and P6 (referred to as bacterial isolates resistant to 4 antimicrobial agents; FOX, AMP, CTX, and SXT) were primarily found in *S. aureus* resistant isolates, 12% each (Table 2).

**Table 2:** Antimicrobial resistance patterns of *S. aureus* isolates

Pattern code	Antimicrobial resistance pattern	Isolates nos.	Pattern (%)	Classes of antibiotics
P1	FOX, AMP	10, 12,17, 19, 20, 27	6 (12%)	2nd generation cephalosporins, penicillins
P2	FOX, AMP, NIT	16	1 (2%)	2nd generation cephalosporins, penicillins, nitrofurantoin
P3	FOX, AMP, AZM	23	1 (2%)	2nd generation cephalosporins, penicillins, macrolide
P4	FOX, AMP, SXT	38	1 (2%)	2nd generation cephalosporins, penicillins, sulfonamides and folic acid inhibitors
P5	FOX, AMP, CTX	44	1 (2%)	2nd generation cephalosporins, penicillins, 3rd generation cephalosporin
P6	FOX, AMP, CTX, SXT	1, 34, 36, 42, 43, 48	6 (12%)	2nd generation cephalosporins, penicillins, 3rd generation cephalosporin, sulfonamides and folic acid inhibitors
P7	FOX, AMP, CTX, NIT	3	1 (2%)	2nd generation cephalosporins, penicillins, 3rd generation cephalosporin, nitrofurantoin
P8	FOX, AMP, CTX, AZM	33, 45	2 (4%)	2nd generation cephalosporins, penicillins, 3rd generation cephalosporin, macrolide
P9	FOX, AMP, SXT, DOX	14	1 (2%)	2nd generation cephalosporins, penicillins, sulfonamides and folic acid inhibitors, tetracycline
P10	FOX, AMP, NIT, DOX	24	1 (2%)	2nd generation cephalosporins, penicillins, nitrofurantoin, tetracycline
P11	FOX, AMP, AZM, DOX	21	1 (2%)	2nd generation cephalosporins, penicillins, macrolide, tetracycline
P12	FOX, AMP, CTX(I), SXT(I), NIT	2	1 (2%)	2nd generation cephalosporins, penicillins, 3rd generation cephalosporin, sulfonamides and folic acid inhibitors, nitrofurantoin
P13	FOX, AMP, CTX(I), SXT, NIT	8	1 (2%)	2nd generation cephalosporins, penicillins, 3rd generation cephalosporin, sulfonamides and folic acid inhibitors, nitrofurantoin
P14	FOX, AMP, CTX, SXT, DOX	15	1 (2%)	2nd generation cephalosporins, penicillins, 3rd generation cephalosporin, sulfonamides and folic acid inhibitors, tetracycline
P15	FOX, AMP, CTX, SXT(I), AZM	47	1 (2%)	2nd generation cephalosporins, penicillins, 3rd generation cephalosporin, sulfonamides and folic acid inhibitors, macrolide
P16	FOX, AMP, CTX(I), SXT, DOX, NIT	18, 49, 37	3 (6%)	2nd generation cephalosporins, penicillins, 3rd generation cephalosporin, sulfonamides and folic acid inhibitors, tetracycline, nitrofurantoin
P17	FOX, AMP, CTX, SXT, DOX, NIT	7	1 (2%)	2nd generation cephalosporins, penicillins, 3rd generation cephalosporin, sulfonamides and folic acid inhibitors, tetracycline, nitrofurantoin
P18	FOX, AMP, CTX, AZM, DOX, NIT	29	1 (2%)	2nd generation cephalosporins, penicillins, 3rd generation cephalosporin, macrolide, tetracycline, nitrofurantoin
P19	FOX, AMP, CTX, AZM, CIP, NIT	4	1 (2%)	2nd generation cephalosporins, penicillins, 3rd generation cephalosporin, macrolide, quinolone, nitrofurantoin
P20	FOX, AMP, CTX(I), GEN, TOB, AZM	50	1 (2%)	2nd generation cephalosporins, penicillins, 3rd generation cephalosporin, aminoglycoside, macrolide
P21	FOX, AMP, CTX(I), GEN, TOB, AMK(I)	32	1 (2%)	2nd generation cephalosporins, penicillins, 3rd generation cephalosporin, aminoglycoside
P22	FOX, AMP, CTX(I), AZM, DOX, CIP	13	1 (2%)	2nd generation cephalosporins, penicillins, 3rd generation cephalosporin, macrolide, tetracycline, quinolone
P23	FOX, AMP, CTX, GEN, TOB, AMK, AZM	41	1 (2%)	2nd generation cephalosporins, penicillins, 3rd generation cephalosporin, aminoglycoside, macrolide
P24	FOX, AMP, CTX(I), AMK, GEN, TOB, AZM	26	1 (2%)	2nd generation cephalosporins, penicillins, 3rd generation cephalosporin, aminoglycoside, macrolide
P25	FOX, AMP, CTX(I), GEN, TOB, AZM, NIT	9	1 (2%)	2nd generation cephalosporins, penicillins, 3rd generation cephalosporin, aminoglycoside, macrolide, nitrofurantoin
P26	FOX, AMP, AMK, GEN, TOB, AZM, SXT	39	1 (2%)	2nd generation cephalosporins, penicillins, aminoglycoside, macrolide, sulfonamides and folic acid inhibitors
P27	FOX, AMP, CTX, SXT(I), AZM, DOX, NIT	6	1 (2%)	2nd generation cephalosporins, penicillins, 3rd generation cephalosporin, sulfonamides and folic acid inhibitors, macrolide, tetracycline, nitrofurantoin
P28	FOX, AMP, CTX(I), CIP, NOR(I), SXT(I), DOX	31	1 (2%)	2nd generation cephalosporins, penicillins, 3rd generation cephalosporin, quinolone, sulfonamides and folic acid inhibitors, tetracycline
P29	FOX, AMP, AMK, GEN, TOB, AZM, SXT, DOX	22	1 (2%)	2nd generation cephalosporins, penicillins, aminoglycoside, macrolide, sulfonamides and folic acid inhibitors, tetracycline
P30	FOX, AMP, CTX, GEN, TOB, AMK(I), AZM, NIT	35	1 (2%)	2nd generation cephalosporins, penicillins, 3rd generation cephalosporin, aminoglycoside, macrolide, Nitrofurantoin
P31	FOX, AMP, CTX(I), GEN, TOB, AMK, AZM(I), DOX	11	1 (2%)	2nd generation cephalosporins,penicillins, 3rd generation cephalosporin, aminoglycoside, macrolide, tetracycline
P32	FOX, AMP, CTX(I), TOB(I), GEN(I), AMK(I) AZM, DOX	46	1 (2%)	2nd generation cephalosporins, penicillins, 3rd generation cephalosporin, aminoglycoside, macrolide, tetracycline
P33	FOX, AMP, CTX(I), CN, TOB, AMK,AZM, NIT	30	1 (2%)	2nd generation cephalosporins, penicillins, 3rd generation cephalosporin, aminoglycoside, macrolide, nitrofurantoin
P34	FOX, AMP, CTX(I), AMK, GEN, TOB, AZM, SXT, DOX	25	1 (2%)	2nd generation cephalosporins, penicillins, 3rd generation cephalosporin, aminoglycoside, macrolide, Sulfonamides and folic acid inhibitors, tetracycline
P35	AMP, FOX, CTX(I), GEN, TOB, NOR, CIP, SXT, AZM	28	1 (2%)	penicillins, 2nd generation cephalosporins, 3rd generation cephalosporin, aminoglycoside, quinolones, sulfonamides and folic acid inhibitors, macrolide
P36	FOX, AMP, CTX(I), TOB(I), GEN(I), AMK(I) AZM, DOX, NIT	5	1 (2%)	2nd generation cephalosporins, penicillins, 3rd generation cephalosporin, aminoglycoside, macrolide, tetracycline, nitrofurantoin
P37	FOX, AMP, CTX(I), AMK, GEN, TOB, AZM, SXT, DOX, NIT	40	1 (2%)	2nd generation cephalosporins, penicillins, 3rd generation cephalosporin, aminoglycoside, macrolide, sulfonamides and folic acid inhibitors, tetracycline, nitrofurantoin

Cefoxitin (FOX), ampicillin (AMP), cefotaxime (CTX), ciprofloxacin (CIP), norfloxacin (NOR), amikacin (AMK), tobramycin (TOB), gentamicin (GEN), doxycycline (DOX), azithromycin (AZM), nitrofurantoin (NIT), sulfamethoxazole/trimethoprim (SXT) and intermediate resistance (I)

### MRSA identification and screening of different enterotoxin genes by conventional PCR

**Fig. 2:**
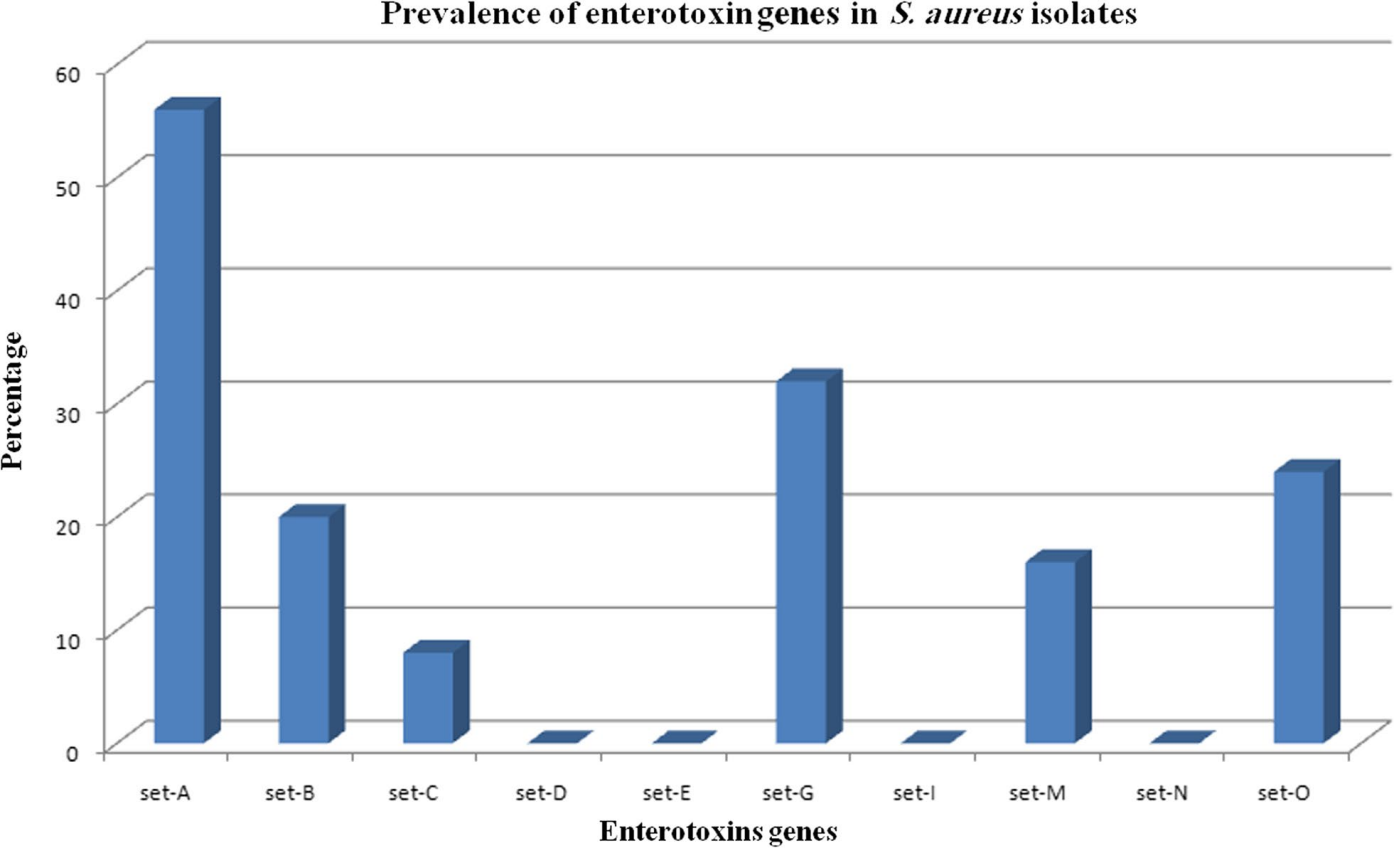
Prevalence of enterotoxin genes in *S. aureus* isolates

**Fig. 3:**
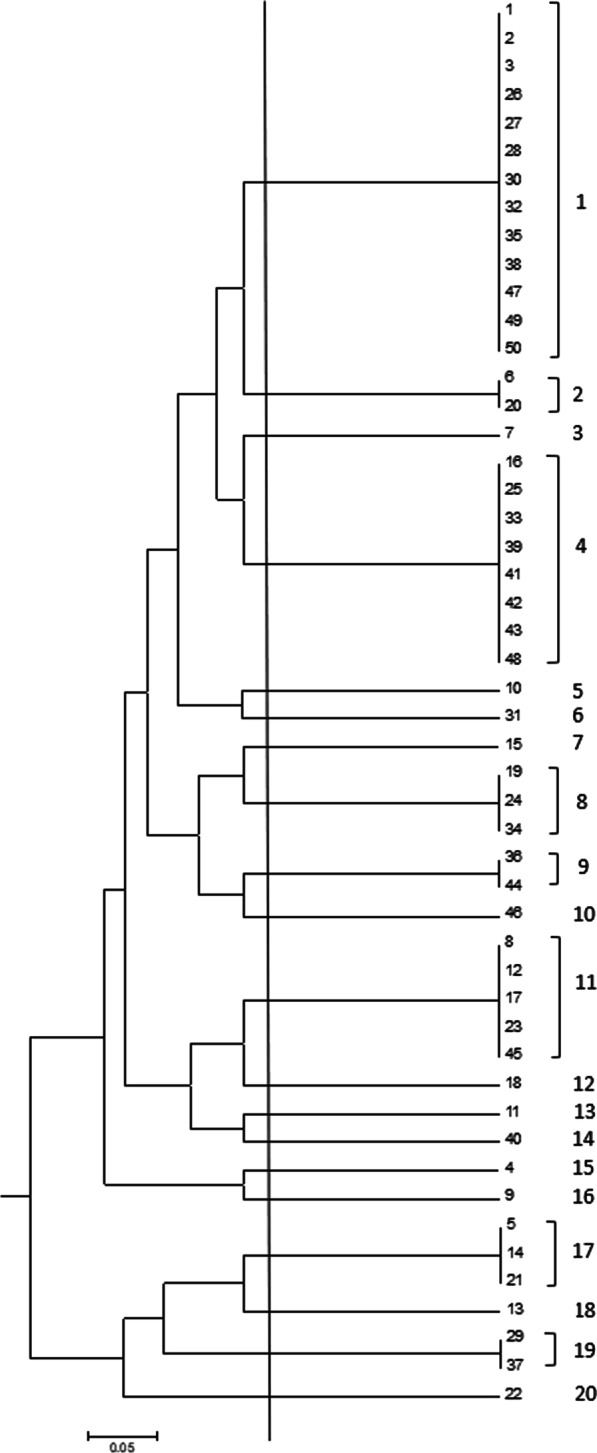
Dendrogram analysis of DNA fingerprinting indicating the diversity of *set* genes among isolates of this study, and categorized into 20 distinct patterns 1–20

Subsequently, *mecA* was amplified using PCR to confirm MRSA identification. All of our isolates tested positive for *mecA*, with a single band at 310 bp. Additionally, PCR was used for *set* genes detection, where *set-A* gene was detected in 56%, while *set-G* gene was detected in 32% of the investigated isolates. In contrast, *set-C* and *set-B* were only detected in 8% and 20% of instances, respectively. Similarly, *Set-M* and *set-O* were detected in 16% and 24% of the tested isolates, respectively (Table 3 and Fig. 2). To classify our isolates, according to the number of *set* genes, identified in each isolate, a correlation analysis (dendrogram) was constructed. The cluster analysis of *set* genes revealed that according to their frequency, they could be categorized into 20 distinct patterns 1–20. Group 1 contained the most prevalent genotype (26%), with only *mecA* identified, followed by group 4 (16%) carrying 2 genes (*mecA* and *set-A*) and group 11 (12%), with 3 genes (*mecA, set-A*, and *set-G*) (Fig. 3).

**Table 3:** Staphylococcal enterotoxin genes pattern groups among the MRSA isolates

Group code		Isolates no.
1	*mecA*	1,2,3,26,27,28,30,32, 35, 38, 47, 49, 50 (26%)
2	*mecA, set-G*	6, 20 (4%)
3	*mecA, set-A, set-M*	7 (2%)
4	*mecA, set-A*	16, 25,33,39,41,42,43,48 (16%)
5	*mecA, set-O*	10 (2%)
6	*mecA, set-A, set-O*	31 (2%)
7	*mecA, set-A, set-B, set-G*	15 (2%)
8	*mecA, set-A, set-B*	19, 24,34 (6%)
9	*mecA, set-B*	36,44 (4%)
10	*mecA, set-B, set- G*	46 (2%)
11	*mecA, set-A, set-G*	8, 17, 12, 23, 4, 45 (12%)
12	*mecA, set-A, set-G, set- O*	18 (2%)
13	*mecA, set-A, set-C, set-G, set-O*	11 (2%)
14	*mecA, set-A,set-C, set-G*	40 (2%)
15	*mecA, set-C, set-O*	4 (2%)
16	*mecA, set-C*	9 (2%)
17	*mecA, set-A, set-G, set-M, set-O*	5, 14, 21 (6%)
18	*mecA, set-A, set-M, set-O*	13 (2%)
19	*mecA, set-A, set-B, set-M, set-O*	37, 29 (4%)
20	*mecA, set-B, set-G, set-M, set-O*	22 (2%)

N.B.: *set-N, set-I, set-D,* and *set-E* are negative in all isolates (50 isolates)

### Validation of microarray by enterotoxin genes carrying isolates

**Fig. 4:**
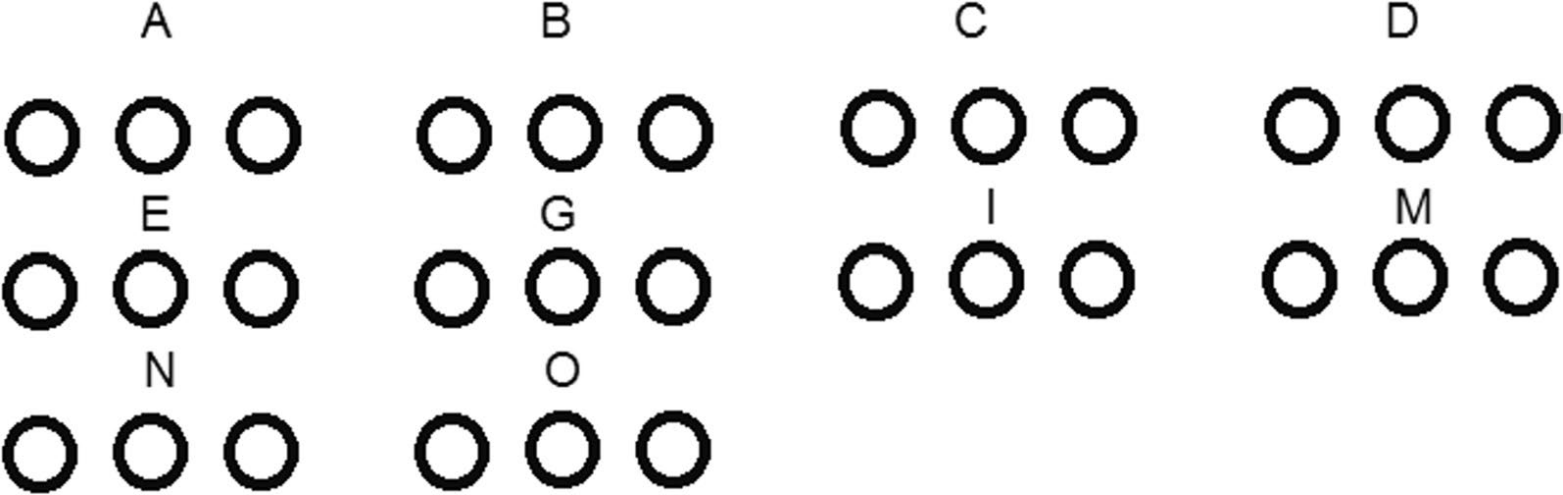
Schematic representation of enterotoxins arrangement in triplicates on each DNA microarray slide including 10 different probes for detection of A, B, C, D, E, G, I, M. N and O SEs genes

**Fig. 5:**
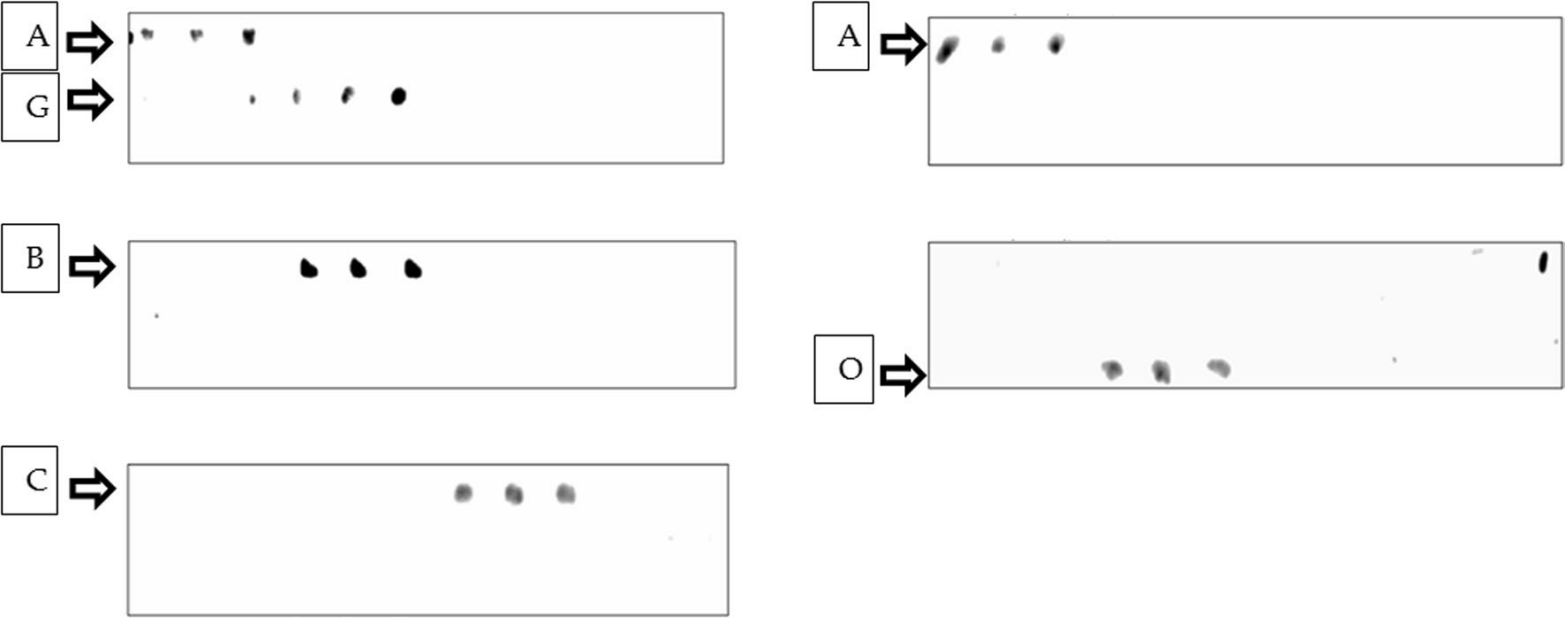
Results of hybridization with the target food poisoning sample DNAs; positive hybridization results were obtained for SEs genes A, B, C, G, and O, on the other hand SEs genes*; set-D, set-E, set-I, set-M*, and *set-N* couldn’t be detected, the same results were obtained by PCR techniques indicating parallel results

The possibility of detecting enterotoxin genes by enterotoxin specific microarray, a more rapid method capable of detecting many different genes simultaneously, was examined. Five different isolates carrying *set-A, set-B, set-C, set-G*, and *set-O* genes were selected for microarray validation. As a result, oligonucleotides used in the microarray test were able to detect *S. aureus* isolates carrying the corresponding genes, indicating the possibility of using the microarray test to detect previously PCR-identified enterotoxins A, B, C, G, and O. (Figs. 4, 5).

## Discussion

MRSA has been called a "superbug" due to its widespread resistance to commonly used antibiotics, making it hard to be treated. The medical complexity of *S. aureus*, including MRSA, arises from the extensive resistance to routinely used medications. Consequently, antibiotic resistance testing is clinically important [31]. Additionally, *S. aureus* is commonly associated with a broad spectrum of human tissue infections and food poisoning, with over 30 different infection causing serotypes ranging from mild to severe systemic infections and sometimes potentially fatal [32]. *S aureus* has the ability to produce many exotoxins, with some strains producing a family of pyrogenic toxins including enterotoxins [33].

Epidemiologically, the production of SEs is a key concern in staphylococcal food poisoning [34]. In addition to their status as superantigens, the staphylococcal enterotoxins have severe negative effects on health when ingested, as these exotoxins in food result in intoxication, violent vomiting, diarrhea, fever, and general symptoms, including headache and nausea, which are all signs of staphylococcal food poisoning [35]. These observations could provide valuable insights into the evolution of *S. aureus* as a pathogen for developing food safety control techniques [34].

During this study, 50 clinical GIT isolates, identified as *S. aureus,* were isolated. In addition, 34% of all cases were < 19 years old, 40% were between 19 and 40 years old, 12% were between 41 and 60 years old, and 14% were > 60 years old. These findings demonstrated that 19-year-olds were more susceptible to infection with *S. aureus,* which is consistent with a previous study in the USA [36]. In addition, SEs were detected in contaminated food, causing staphylococcal food poisoning, toxic shock, and allergic and autoimmune reaction [37].

In this study, MDR was identified in 43 (86%) isolates. The current results were consistent with those reported in China [38], where approximately 67% of the *S. aureus* isolates were MDR. In contrast, a lower proportion was reported in another study in China [39], where 57.5% of their tested *S. aureus* isolates were resistant to three or more classes of antimicrobials (MDR).

In this study, antimicrobial susceptibility testing revealed that all isolates were resistant to both ampicillin and cefoxitin (100%), followed by cefotaxime (72%). These results exceeded those previously reported in China [38], demonstrating that 92% of *S. aureus* isolates were penicillin-resistant, whereas only 10% were cefoxitin-resistant. Regarding two antimicrobials, i.e., nitrofurantoin and gentamicin, only 34% and 30% of the tested isolates were identified as resistant, respectively. However, much lower percentages for both nitrofurantoin (5%) and gentamicin (2%) were previously reported in China [38] (Table 2).

In this study, 36% and 8% of the tested isolates exhibited resistance to doxycycline and ciprofloxacin, respectively. However, other different values for doxycycline and ciprofloxacin were previously reported in Iran [33], where 12% and 18% of all isolates were identified as doxycycline and ciprofloxacin resistant, respectively. In this study, similar to ciprofloxacin, a very high sensitivity (100%) was confirmed against vancomycin and linezolid. In case of other antimicrobials, a higher level of resistance to cefotaxime (72%) was reported among the isolates in this study. Similarly, a close result for cefotaxime resistance (71.4%) was detected in another study in Iran [40]. For the identification of enterotoxin specificity and diversity, SE gene patterns of *S. aureus* isolates from patients suffering from food poisoning were analyzed. In addition, the *mecA* gene was detected in each of the tested isolates, indicating its contribution to their resistance [41].

Cefoxitin disc diffusion method was used to validate the phenotypic MRSA identification [42]. Interestingly, the frequency of the *mecA* gene among the examined *S. aureus* was equal to 100%. As mentioned previously, cefoxitin disc represents a useful tool for predicting methicillin resistance, with sensitivity values approaching 100% [43]. Moreover, identifying the *mecA* gene by PCR can be used as a positive indicator for MRSA isolates [44].

In this study, each clinical isolate tested was *mecA*-positive. This result was consistent with another study in Egypt [31], in which 100% of *S. aureus* were MRSA isolates. In the same vein, high rates of MRSA were reported in Nepal [45] and Eritrea (72%) [46]. However, moderate rates (56%) were reported in Romania [47]. In addition, the reported results from this study showed a higher prevalence of MRSA than those reported in other studies in Sweden and India, respectively [48, 49], in which variable percentages ranging from 44 to 81% were detected, respectively. This variation may be attributed to different antibiotic prescribing strategies across nations. Although methicillin was the first semisynthetic penicillinase-resistant penicillin identified, it was withdrawn from the market in the United States because of the high incidence of interstitial nephritis associated with its use [50]. In this study, *mecA*-positive strains demonstrated antimicrobial resistance to cefoxitin, validating the use of the cefoxitin disc as reported previously [51].

The study investigated the prevalence of various enterotoxin genes using conventional PCR. Traditional SEs, such as A, B, C, D, and E, in addition to other SEs, such as G, I, M, N, and O, commonly detected in previous studies, were selected. According to the previous results obtained, several enterotoxin genes were detected in the tested strains of *S. aureus*, particularly including *set-A, set-B, set-C, set-D, set-E set-G, set-I*, *set-M, set-N*, and *set-O* [6, 52]. In this study, *set-A* exhibited the highest prevalence at 56%, followed by *set-G*, *set-O*, and *set-B* at 32%, 24%, and 20%, respectively. Nonetheless, 16% and 8% were discovered in *set M* and *set C,* respectively. S*et-D, set-E, set-I*, and *setN* were not detected in any of the tested isolates. The study results were higher than those found in a study conducted in Tanzania, where enterotoxin C and B genes were detected in approximately 0.3% of the tested isolates, whereas *set-A* was completely absent [53]. Similarly, genes for both enterotoxins D and A were detected in approximately 10% of isolates in Turkey [54]. In contrast, a study in Sudan [14] reported that none of the enterotoxin genes was present in their isolates. This variation between the results of different studies may be attributable to several factors, such as the geographical origin, the source and size of the tested samples, and the type and sensitivity of the chosen method for detecting these genes.

The cluster analysis of *set* genes revealed that group 1 was the most prevalent genotype without any of the *set* genes detected, followed by group 4 carrying *set-A* gene. However, in the case of isolates carrying more than 2 *set* genes, group 11 was the most prevalent with both *set-A* and *set-G* genes. Moreover, the results obtained indicated that isolates carrying more than 2 *set* genes were rarely detected.

As previously reported, microarray and PCR produced similar results, making microarray testing for enterotoxins an alternative tool to be validated in this study [30]. In microarray experiments, DNA probes were immobilized at high density carrying more than one copy of each target gene in each DNA chip, which increased the possibility of carrying out each experiment in triplicates or more on the same slide. In addition, DNA microarrays were used primarily in mixed microbial community analyses based on ribosomal DNA sequence [55, 56]. Moreover, other microarray types could be used to detect some virulence factors [57].

In this study, oligonucleotides in the microarray test were able to detect the corresponding genes, indicating the possibility of using the microarray as a reliable method for detecting and identifying many SEs in replicates on the same slide.

## Conclusion

In conclusion, it could be reported that the genotypic findings of this study may help distinguish the common types of enterotoxigenic *S. aureus* among Egyptian patients with food poisoning. They could be beneficial for the study and management of *S. aureus* infections in food.

## Data Availability

All relevant data are available upon request from the corresponding author.
